# Solving large break minimization problems in a mirrored double round-robin tournament using quantum annealing

**DOI:** 10.1371/journal.pone.0266846

**Published:** 2022-04-08

**Authors:** Michiya Kuramata, Ryota Katsuki, Kazuhide Nakata

**Affiliations:** 1 Department of Industrial Engineering and Economics, Tokyo Institute of Technology, Tokyo, Japan; 2 NTT DATA Corporation, Tokyo, Japan; Eötvös Loránd University, HUNGARY

## Abstract

Quantum annealing has gained considerable attention because it can be applied to combinatorial optimization problems, which have numerous applications in logistics, scheduling, and finance. In recent years, with the technical development of quantum annealers, research on solving practical combinatorial optimization problems using them has accelerated. However, researchers struggle to find practical combinatorial optimization problems, for which quantum annealers outperform mathematical optimization solvers. Moreover, there are only a few studies that compare the performance of quantum annealers with the state-of-the-art solvers, such as Gurobi and CPLEX. This study determines that quantum annealing demonstrates better performance than the solvers in that the solvers take longer to reach the objective function value of the solution obtained by the quantum annealers for the break minimization problem in a mirrored double round-robin tournament. We also explain the desirable performance of quantum annealing for the sparse interaction between variables and a problem without constraints. In this process, we demonstrate that this problem can be expressed as a 4-regular graph. Through computational experiments, we solve this problem using our quantum annealing approach and two-integer programming approaches, which were performed using the latest quantum annealer D-Wave Advantage, and Gurobi, respectively. Further, we compare the quality of the solutions and the computational time. Quantum annealing was able to determine the exact solution in 0.05 seconds for problems with 20 teams, which is a practical size. In the case of 36 teams, it took 84.8 s for the integer programming method to reach the objective function value, which was obtained by the quantum annealer in 0.05 s. These results not only present the break minimization problem in a mirrored double round-robin tournament as an example of applying quantum annealing to practical optimization problems, but also contribute to find problems that can be effectively solved by quantum annealing.

## Introduction

Quantum annealing [1] can be applied to combinatorial optimization problems, which have numerous applications in logistics, scheduling, and finance. Quantum annealers, which are hardware devices that perform quantum annealing, are relatively noise resistant and consist of an increasing number of quantum bits (qubits). In 2017, D-Wave Systems released a quantum annealer with 2048 qubits, and have now released one with 5760 qubits [2]. Although several problems [3] remain unresolved, the increase in the qubits and reduction in the noise of the quantum annealer are among the primary advancements [4]. In recent years, quantum annealers have been used to solve practical combinatorial optimization problems [5–10]. However, researchers struggle to find practical combinatorial optimization problems, for which quantum annealers outperform other mathematical optimization solvers. Moreover, only a few studies [9, 11] have compared the performance of quantum annealers with mathematical optimization solvers, such as Gurobi [12] and CPLEX [13]. One of the fields that quantum annealing can be applied is sports scheduling. We thus considered this field to perform our comparison between quantum annealing and other solvers.

Sports scheduling involves the construction of a suitable schedule for sports competitions. It has several practical constraints and splits into various combinatorial optimization problems [14, 15]. Among them, in a round-robin tournament (RRT), where each team plays against every other team once, in a double round-robin tournament (DRRT), where each team plays against the others twice, and in a mirrored double round-robin tournament (MDRRT), which is a DRRT with the same combination of games in the first and second halves, much research has been conducted on the break minimization problem, which determines whether the game is held in the venue of the team, or its opponent [15–21]. RRTs, DRRTs, and MDRRTs are adopted in many professional sports, such as soccer and basketball [15, 22–25]. The break minimization problem is derived from practical requirements, and is known to be very difficult to solve. To address this problem, methods such as integer programming [16, 18] and constraint programming [17] have been developed in the past.

The contributions of this study are as follows: First, we found that the break minimization problem in an MDRRT is a practical combinatorial optimization problem, for which quantum annealing demonstrates better performance than the mathematical optimization solvers in that the solvers take longer to achieve the objective function values of the solutions obtained by the quantum annealers. Second, we explain that the break minimization problem is easy to solve using quantum annealers because of the sparse interaction between variables and the lack of constraints. In this process, we demonstrate that the break minimization problem in an MDRRT can be expressed as a 4-regular graph. Third, we solve this problem using the latest quantum annealer D-Wave Advantage, and one of the most sophisticated mathematical optimization solvers, Gurobi, and compare the quality of their solutions through computational experiments. We also measure the time it takes for Gurobi to reach the objective function value, which the quantum annealer reaches in 0.05 s.

## Quantum annealing using D-Wave Advantage

Quantum annealing [1] is a method for solving combinatorial optimization problems using quantum fluctuations. The solution of the combinatorial optimization problem is obtained by first applying a strong transverse field, and then gradually weakening the transverse field. This is similar to simulated annealing [26]; however, quantum annealing is performed using a quantum annealer as a physical phenomenon, whereas simulated annealing is calculated using a classical computer. Quantum annealing minimizes the energy of the Ising model (Eq (1)), associated with the combinatorial optimization problem.

$$\begin{array}{c} E=\sum_{i,j}J_{ij}s_{i}s_{j}+\sum_{i}h_{i}s_{i},\;s_{i}=\pm 1,\;\;\forall i\in V. \end{array}$$ (1)

In Eq (1), *J*_*i*,*j*_ is the coupling strength between the *i*th and *j*th spin, and *h*_*i*_ is the bias. *V* is a set of spins, and each spin *s*_*i*_ takes the value 1 or −1. By defining *s*_*i*_ = 2*x*_*i*_ − 1 in Eq (1), we obtain quadratic unconstrained binary optimization (QUBO), which is suitable for representing combinatorial optimization problems. QUBO is defined as Eq (2).

$$\begin{array}{c} \begin{array}{cc} \text{minimize} & q^{⊤}Qq\\ \text{subject}\;\text{to} & q\in {\left\{0,1\right\}}^{L}. \end{array} \end{array}$$ (2)

In Eq (2), *q*_*i*_ is a binary variable, and *L* is the number of binary variables. $Q\in R^{L\times L}$ is a matrix that characterizes the combinatorial optimization problem. QUBO is a problem that determines the values of **q** that minimize **q**^⊤^ *Q* **q**. Because this QUBO is equivalent to the Ising model, we can solve the combinatorial optimization problem on a quantum annealer.

**Fig 1:**
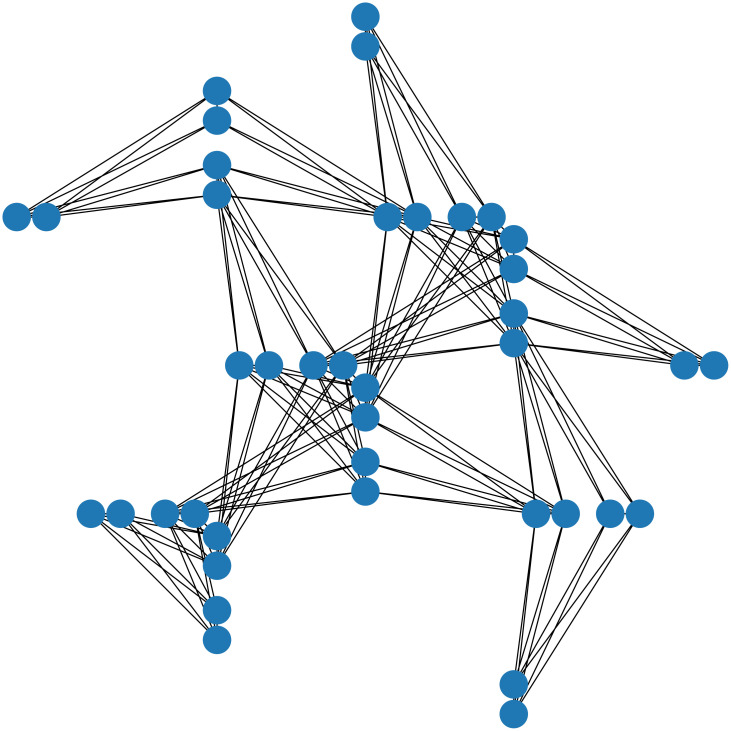
Pegasus graph. The blue circles represent the qubits and the solid black lines represent the connections between the qubits.

However, to solve the QUBO transformed into the Ising model using the quantum annealer, a minor-embedding is necessary. Minor embedding refers to associating the Ising model with qubits in the quantum processing unit topology (Fig 1). Fig 1 is a Pegasus graph mounted on D-Wave Advantage, a quantum annealer with 5760 qubits. In Fig 1, the blue circles represent the qubits and the solid black lines represent the connections between the qubits. As shown in Fig 1, because the connectivity between qubits is sparse, multiple qubits may be required to represent one logical variable. In particular, QUBO with many non-zero elements of *Q* in Eq (2) consumes many qubits to embed it in a quantum annealer. This is difficult in practice; however, D-Wave Systems have resolved the problem. The maximum degree of the Chimera graph, the hardware graph made by D-Wave Systems, is 6. The maximum degree of the Pegasus graph, the more recent hardware graph, is 15 [2]. Therefore, it is easier to represent combinatorial optimization problems in a Pegasus graph, than in a Chimera graph.

## Break minimization problem on a mirrored double round-robin tournament

### Definition of the problem

An RRT is a competition that meets the following conditions.

1. Each team meets every other team once.
2. Each team has its own venue in its home town. A home game for a team is an away game for its opponent.
3. Each game is played at the home of either of the teams, or its opponent.

A DRRT is a competition that meets the following four conditions.

1. Each team meets every other team twice.
2. Each team has its own venue in its home town.
3. Each game is played at the home of either the team or its opponent.
4. If the first game against an opponent is played at the team’s home (/ away) venue, then the second game is played at the away (/ home) venue.

An MDRRT is a DRRT where the first half is the same as the second half, except for exchanging the home games and away games.

We define the symbols. The timetable shows the opponents of all teams and their slots. The slots refer to dates. A home-away assignment (HA-assignment) defines which team’s home ground, the game will be hosted at. The symbols used in the mathematical expressions are as follows.

- 2*n*: the number of teams. *n* is an integer of 2 or greater.
- *T* ∈ {1, 2, …, 2*n*}: a set of teams.
- *S* ∈ {1, 2, …, 2(2*n* − 1)}: a set of slots.
- *τ*(*t*, *s*) ∈ *T* × *S*: the opponent that plays against team *t* at slot *s*.
-
T : a timetable. The (*t*, *s*) entry of T is *τ*(*t*, *s*).
- *a*(*t*, *s*): *a*(*t*, *s*) = 1 if team *t* plays against team *τ*(*t*, *s*) in slot *s* at team *t*’s home, *a*(*t*, *s*) = 0 otherwise.
-
A : this represents an HA-assignment. The (*t*, *s*) entry of A is *a*(*t*, *s*).

Table 1 is a timetable T for *n* = 2. In Table 1, the vertical axis represents the teams and the horizontal axis represents the slots. Each entry (*t*, *s*) of the timetable T is an opponent *τ*(*t*, *s*). Because Table 1 is the timetable for the MDRRT, *τ*(*t*, *s*) = *τ*(*t*, *s* + 2*n* − 1) (∀(*t*, *s*) ∈ *T* × {1, …, 2*n* − 1}) holds. Table 2 shows the HA-assignment A corresponding to the timetable in Table 1. Each entry (*t*, *s*) of A indicates whether a game in which team *t* plays against team *τ*(*t*, *s*) in slot *s* is a home game or an away game. The game between team *t* and team *τ*(*t*, *s*) in slot *s* is held at the home of team *t* if *a*(*t*, *s*) = 1 and at the home of team *τ*(*t*, *s*) if *a*(*t*, *s*) = 0. A break means that a team plays at two home games or two away games in a row. For example, in Table 2, Team 2 has a break in slot 2 because of the two consecutive away games. Similarly, Team 2 has a break in slot 4 because of the two consecutive home games. Breaks should be avoided as much as possible to ensure fairness among teams.

**Table 1:** Timetable.

Slot	1	2	3	4	5	6
**team 1**	2	3	4	2	3	4
**team 2**	1	4	3	1	4	3
**team 3**	4	1	2	4	1	2
**team 4**	3	2	1	3	2	1

**Table 2:** Home away assignment.

Slot	1	2	3	4	5	6
**team 1**	1	0	1	0	1	0
**team 2**	0	0	1	1	1	0
**team 3**	1	1	0	0	0	1
**team 4**	0	1	0	1	0	1

As described in [22] and [23], in practical application, various constraints must be considered in scheduling sports tournaments. Further, Régin [17] divided the scheduling in an RRT into a first stage, where many practical constraints are involved, and a second stage, where there are no constraints; the first and second stages are as follows.

- Considering various constraints, the schedule is created without deciding the assignment of the venue.
- Each game is assigned to either the home game or away game.

This approach is useful when there are many constraints in the creation of a schedule. The second stage corresponds to the break minimization problem. In this study, the break minimization problem in an MDRRT is defined as follows, similar to [27].

#### Break minimization problem in an MDRRT

**INPUT**: A timetable in an MDRRT without an HA-assignment.

**TASK**: Find an HA-assignment with the smallest number of breaks for a given timetable.

Trick [16] points out that solving the break minimization problem is more difficult than determining the timetable corresponding to the first stage. In this study, we focus on solving the break minimization problem, similar to Régin [17] and Trick [16].

### Previous studies

The break minimization problem in an RRT has been studied by many researchers [16, 18, 20]. In particular, De Werra and Dominique [28] proved that the number of breaks in an RRT is more than 2*n* − 2, and the number of breaks in an MDRRT is more than 6*n* − 6. As explained in Section “Definition of the problem”, 2*n* is the number of teams. Although these properties were clarified, it is very difficult to solve the break minimization problem in an RRT, a DRRT, and an MDRRT. To solve this problem, constraint programming [17], integer programming [16, 18], and an approximation algorithm [20] have been studied in the past. In recent years, Urdaneta *et al*. [18] demonstrated, through numerical experiments that formulating the problem as an unconstrained quadratic integer programming problem and solving it using the mathematical optimization solver is superior to other formulations with constraints. In this section, we introduce the study by Urdaneta *et al*. [18].

Before describing formulations in [18], we explain certain symbols required in the formulations.

$$\begin{array}{c} K\left(k\right)=\{\left(t_{k},s_{k}\right),\left(t_{k},s_{k}^{{}^{\prime }}\right),\left(t_{k}^{{}^{\prime }},s_{k}\right),\left(t_{k}^{{}^{\prime }},s_{k}^{{}^{\prime }}\right)\} \end{array}$$ (3)

In Eq (3), K(k) represents the games and their slots between the two teams of the *k*th combination. *k* can be any integer between 1 and (2n2). *t*_*k*_ and $t_{k}^{\prime }$ represent the two teams in the *k*th combination, respectively. The slot of the first game between *t*_*k*_ and $t_{k}^{\prime }$ is *s*_*k*_, and the slot of the second game is $s_{k}^{\prime }$. For any *k*, Eq (4) holds.

$$\begin{array}{c} \left|K\left(k\right)\right|=4,\;k\neq k^{{}^{\prime }}\Rightarrow K\left(k\right)\cap K\left(k^{{}^{\prime }}\right)=\emptyset ,\;T\times S=\overset{n(2n-1)}{\underset{k=1}{⋃}}K\left(k\right). \end{array}$$ (4)

|K(k)|=4 indicates that K(k) contains four games in the timetable. $k\neq k^{{}^{\prime }}\Rightarrow K\left(k\right)\cap K\left(k^{{}^{\prime }}\right)=\emptyset$ also indicates that two different K(k) do not contain the same games. $T\times S={⋃}_{k=1}^{n(2n-1)}K\left(k\right)$ indicates that adding up all the combinations equals the original timetable.

The binary variable *y*_*ts*_ takes 1 if team *t* plays at home in slot *s* and 0 if it plays away. Eq (5) holds for any *k* ∈ {1, 2, …, *n*(2*n* − 1)} to satisfy the conditions of the DRRTs.

$$\begin{array}{c} \begin{array}{c} y_{t_{k}s_{k}}+y_{t_{k}^{\prime }s_{k}}=1\\ y_{t_{k}s_{k}}+y_{t_{k}s_{k}^{\prime }}=1\\ -y_{t_{k}s_{k}}+y_{t_{k}^{\prime }s_{k}^{\prime }}=0 \end{array} \end{array}$$ (5)

The first constraint is that each team plays at its home or at its opponent’s home. The second constraint implies that a team plays at home (/ away) against its opponent first, and then plays away (/ home) against its opponent second. The third implies that the first game is played at the home (/ away) of the team, and the second game is played at the home (/ away) of the opponent. These represent the second, third, and fourth of the four conditions for a DRRT, respectively.

Urdaneta *et al*. did not provide a specific formulation for the break minimization problem. They demonstrated that the constrained optimization problem (Eq (6)), which represents a break minimization problem in a DRRT, can be expressed as an unconstrained optimization problem (Eq (8)).

$$\begin{array}{c} \begin{array}{cc} \text{minimize} & l\left(y\right)=c^{⊤}y+\frac{1}{2}y^{⊤}Hy\\ \;\text{subject}\;\text{to}\; & y_{t_{k}s_{k}}+y_{t_{k}^{\prime }s_{k}}=1\;\left(\forall k\in \left\{1,2,\ldots ,n\left(2n-1\right)\right\}\right),\\ & y_{t_{k}s_{k}}+y_{t_{k}s_{k}^{\prime }}=1\;\left(\forall k\in \left\{1,2,\ldots ,n\left(2n-1\right)\right\}\right),\\ & -y_{t_{k}s_{k}}+y_{t_{k}^{\prime }s_{k}^{\prime }}=0\;\left(\forall k\in \left\{1,2,\ldots ,n\left(2n-1\right)\right\}\right),\\ & y_{t,s}\in \left\{0,1\right\}\;\left(\forall t\in T,\forall s\in S\right). \end{array} \end{array}$$ (6)

The constraints are the same as in Eq (5). We define *z*_*k*_ as $z_{k}:=y_{t_{k}s_{k}}$ for the first component (*t*_*k*_, *s*_*k*_) of K(k). The variables relating to the components of K(k) can be replaced, as in Eq (7).

$$\begin{array}{c} y_{t_{k}s_{k}}=z_{k},\;y_{t_{k}^{\prime }s_{k}}=1-z_{k},\;y_{t_{k}s_{k}^{\prime }}=1-z_{k},\;y_{t_{k}^{\prime }s_{k}^{\prime }}=z_{k} \end{array}$$ (7)

Thus, substituting the transformation described in Eq (7) into the constrained formulation in Eq (6) yields the unconstrained formulation in Eq (8).

$$\begin{array}{c} \begin{array}{cc} \;\text{minimize}\; & \bar{l}\left(z\right)=\bar{a}+{\bar{c}}^{⊤}z+\frac{1}{2}z^{⊤}\bar{H}z\\ \;\text{subject}\;\text{to}\; & z\in {\left\{0,1\right\}}^{n(2n-1)}. \end{array} \end{array}$$ (8)

In Eq (8), $\bar{a}$, $\bar{c}$, and $\bar{H}$ are obtained by substituting Eq (7) for Eq (6).

## Formulation and analysis of the problem

In Section “Formulating the break minimization problem in a mirrored double round-robin tournament”, we specify the formulation by Urdaneta *et al*. [18] as a break minimization problem in an MDRRT. In Section “Analysis: Benefits of the sparsity of the problem” and “Analysis: Benefits of no constraints”, we show that the problem satisfies certain theoretical and experimental characteristics that make it more suitable for the quantum annealers and may account for their ability to obtain high-quality solutions rapidly.

### Formulating the break minimization problem in a mirrored double round-robin tournament

As mentioned earlier, Urdaneta *et al*. [18] does not provide a specific objective function for Eqs (6) and (8). Further, we set the objective function as the total number of breaks in the MDRRT. Our formulation corresponding to Eq (6) is Eq (9).

$$\begin{array}{c} \begin{array}{cc} \text{minimize} & f\left(y\right)=\sum_{t\in T}\sum_{s\in S\backslash \{4n-2\}}\left(y_{ts}y_{ts+1}+\left(1-y_{ts}\right)\left(1-y_{ts+1}\right)\right)\\ \;\text{subject}\;\text{to}\; & y_{t_{k}s_{k}}+y_{t_{k}^{\prime }s_{k}}=1\;\left(\forall k\in \left\{1,2,\ldots ,n\left(2n-1\right)\right\}\right),\\ & y_{t_{k}s_{k}}+y_{t_{k}s_{k}^{\prime }}=1\;\left(\forall k\in \left\{1,2,\ldots ,n\left(2n-1\right)\right\}\right),\\ & -y_{t_{k}s_{k}}+y_{t_{k}^{\prime }s_{k}^{\prime }}=0\;\left(\forall k\in \left\{1,2,\ldots ,n\left(2n-1\right)\right\}\right),\\ & y_{ts}\in \left\{0,1\right\}\;\left(\forall t\in T,\forall s\in S\right). \end{array} \end{array}$$ (9)

The objective function *f*(**y**) consists of the sum of *y*_*ts*_ *y*_*t*,*s*+1_, which represents a break corresponding to the two consecutive home games and (1 − *y*_*ts*_)(1 − *y*_*ts*+1_), which represents a break corresponding to the two consecutive away games. As Urdaneta *et al*. did, we transform the constrained formulation in Eq (9) into the unconstrained formulation in Eq (10) using the transformation described in Eq (7).

$$\begin{array}{c} \begin{array}{cc} \text{minimize} & \sum_{t_{k}\in T}\sum_{s_{k}\in S\backslash \left\{4n-2\right\}}a\left(t_{k},s_{k}\right)(z_{k}z_{k^{\prime }}+\left(1-z_{k}\right)\left(1-z_{k^{\prime }}\right))\\ & \;+b\left(t_{k},s_{k}\right)(\left(1-z_{k}\right)z_{k^{\prime }}+z_{k}\left(1-z_{k^{\prime }}\right))\\ & \;+c\left(t_{k},s_{k}\right)(z_{k}\left(1-z_{k^{\prime }}\right)+\left(1-z_{k}\right)z_{k^{\prime }})\\ & \;+d\left(t_{k},s_{k}\right)(z_{k}z_{k^{\prime }}+\left(1-z_{k}\right)\left(1-z_{k^{\prime }}\right))\\ \;\text{subject}\;\text{to}\; & z_{k}\in \left\{0,1\right\}\;\left(\forall k\in \left\{1,2,\ldots ,n\left(2n-1\right)\right\}\right). \end{array} \end{array}$$ (10)

In Eq (10), *k*′ satisfies $\left(t_{k},s_{k}+1\right)\in K\left(k^{\prime }\right)$ for any *k*. Let *a*(*t*_*k*_, *s*_*k*_) = 1 if $\left(y_{t_{k}s_{k}}=z_{k}\right)\wedge \left(y_{t_{k}s_{k}+1}=z_{k^{\prime }}\right)$; otherwise, *a*(*t*_*k*_, *s*_*k*_) = 0. Let *b*(*t*_*k*_, *s*_*k*_) = 1 if $\left(y_{t_{k}s_{k}}=1-z_{k}\right)\wedge \left(y_{t_{k}s_{k}+1}=z_{k^{\prime }}\right)$, otherwise *b*(*t*_*k*_, *s*_*k*_) = 0. Let *c*(*t*_*k*_, *s*_*k*_) = 1 if $\left(y_{t_{k}s_{k}}=z_{k}\right)\wedge \left(y_{t_{k}s_{k}+1}=1-z_{k^{\prime }}\right)$, otherwise *c*(*t*_*k*_, *s*_*k*_) = 0. Let *d*(*t*_*k*_, *s*_*k*_) = 1 if $\left(y_{t_{k}s_{k}}=1-z_{k}\right)\wedge \left(y_{t_{k}s_{k}+1}=1-z_{k^{\prime }}\right)$, otherwise *d*(*t*_*k*_, *s*_*k*_) = 0. Eq (10) is the QUBO formulation. Therefore, we transformed Eq (10) into an equivalent Ising model and solved it using quantum annealing. As described in Section “Analysis: Benefits of the sparsity of the problem”, the break minimization problem in an MDRRT is suitable for solving the problem using a quantum annealer.

### Analysis: Benefits of the sparsity of the problem

**Fig 2:**
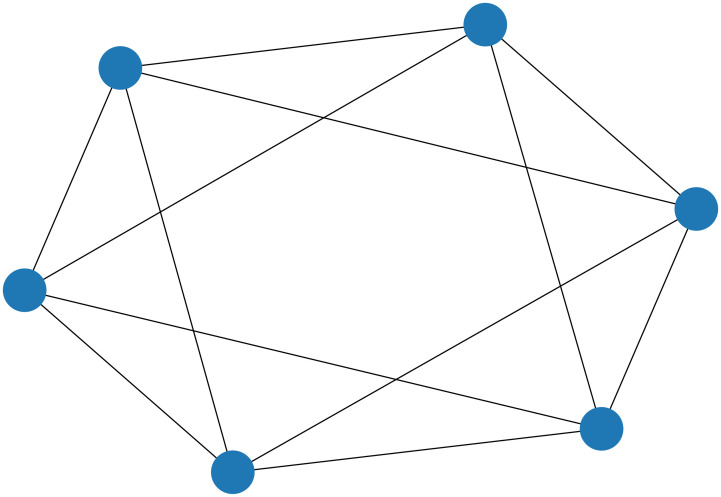
Source graph of the break minimization problem defined by Table 1. The blue nodes represent logical variables, and the solid black lines represent the interaction between variables.

In this section, we show that the break minimization problem in an MDRRT has a sparse graph representation, which makes it more suitable for the quantum annealers. As in [29], we refer to the graph representation of QUBO as the source graph, and the quantum processing unit topology of a quantum annealer as the target graph. The source graph contains nodes that represent logical variables and edges that represent the interaction between variables. The target graph has nodes that represent qubits and edges that represent the connections between the qubits, as shown in Fig 1. Fig 2 shows the source graph of the break minimization problem for the schedule defined in Tables 1 and 2. As can be observed from Fig 2, the source graph of the break minimization problem in an MDRRT is a 4-regular graph.

**Proposition 1** *The source graph of the break minimization problem in an MDRRT is a 4-regular graph*.

*Proof* As shown in Eq (7), the variable *z*_*k*_ represents the four games denoted by $\left\{y_{ts}|\left(t,s\right)\in K\left(k\right)\right\}$. In addition, in an MDRRT, the combination of opponents for the games before and after a certain game is the same for the first and second halves, and thus, *z*_*k*_ is related to the four variables. Therefore, the source graph of a break minimization problem in an MDRRT is a 4-regular graph.

Regardless of the number of teams in an MDRRT, the source graph is a 4-regular graph, which means that the graph is very sparse. However, the source graph in a DRRT is not always a regular graph, and its degree is less than, or equal to 8.

**Proposition 2** *The degree of the source graph of a break minimization problem in a DRRT is less than or equal to 8*.

*Proof* The variable *z*_*k*_ represents the four games denoted by $\left\{y_{ts}|\left(t,s\right)\in K\left(k\right)\right\}$. In contrast to an MDRRT, in a DRRT, the combination of opponents in the games before and after a certain game is not always equal in the first and second halves. Therefore, when the degree of the source graph is the largest, *z*_*k*_ is related to eight variables that represent the before and after of the game in the first and second halves. Thus, the maximum degree of the source graph of the break minimization problem in a DRRT is 8.

In Tables 3 and 4, we demonstrate the minor embeddings of the source graph of the break minimization problem in an MDRRT and a DRRT, respectively. The minor embeddings are in the target graph of the D-Wave Advantage. We perform minor embedding using the method described in [1]. We randomly create five break minimization problems in both, MDRRTs and DRRTs. Tables 3 and 4 show the number of nodes (**Nodes** in the tables) and edges (**Edges** in the tables) in the source graph of the break minimization problem in an MDRRT and a DRRT, the total number of qubits used (**Qubits** in the tables), and the number of qubits used per node in the source graph (**Qubits/Nodes** in the tables). **Teams** in the tables represent the number of teams included in the problems.

**Table 3:** Number of nodes and edges of the source graph and used qubits in MDRRTs.

Teams	Nodes	Edges	Qubits	Qubits/Nodes
4	6	12	8	1.333333
8	28	56	39	1.392857
12	66	132	112	1.696970
16	120	240	209	1.741667
20	190	380	370	1.947368
24	276	552	547	1.981884
28	378	756	755	1.997354
32	496	992	1467	2.957661
36	630	1260	1663	2.639683
40	780	1560	1973	2.529487
44	946	1892	3215	3.398520
48	1128	2256	3975	3.523936

**Table 4:** Number of nodes and edges of the source graph and qubits used in DRRTs.

Teams	Nodes	Edges	Qubits	Qubits/Nodes
4	6	10.4	7.2	1.200000
8	28	83.2	50.8	1.814286
12	66	232.8	212.0	3.212121
16	120	441.6	613.2	5.110000
20	190	676.0	1167.8	6.146316
24	276	998.4	2412.4	8.740580
28	378	1411.2	3878.4	10.260317

In the target graph of D-Wave Advantage, we were able to minor-embed up to 48 teams in an MDRRT, and up to 28 teams in a DRRT. Therefore, up to 48 teams can be considered in Table 3, and up to 28 teams can be considered in Table 4. In Tables 3 and 4, we can observe that the break minimization problem in an MDRRT and a DRRT is sparse. Comparing Tables 3 and 4, we can observe that the source graph in an MDRRT is sparser than that in a DRRT, and the number of qubits used is smaller. In [30], the authors investigated how the degree of the graph of MAX-CUT problems affects the quality of the solution obtained from a quantum annealer, and demonstrated that the smaller the degree of the graph, the better the quality. Thus, it is expected that the break minimization problem in an MDRRT is easier to solve, than in a DRRT, using a quantum annealer. This is also confirmed by the experimental results shown in Tables 8 and 9. The size of the problem that can be solved on the quantum annealer is also larger for an MDRRT than for a DRRT. We were able to solve problems with up to 48 teams in an MDRRT and up to 28 teams in a DRRT, using D-Wave Advantage.

### Analysis: Benefits of no constraints

In this section, we show that the break minimization problem in an MDRRT is an unconstrained optimization problem, which makes it more suitable for the quantum annealers. As shown in Eq (10), the break minimization problem can be expressed naturally as an unconstrained optimization problem. In other words, all solutions searched for by quantum annealing are feasible solutions, and thus, the search conducted using quantum annealing is efficient. However, for problems with hard constraints, such as quadratic assignment problems [31] and traveling salesman problems [32], many of the solutions explored using quantum annealing are deemed infeasible. As an example, we consider an optimization problem with severe constraints (Eq (11)), such as the quadratic assignment problem or traveling salesman problem, wherein the objective function is assumed to return 0 regardless of the solution.

$$\begin{array}{c} \begin{array}{cc} \text{minimize} & 0\\ \;\text{subject}\;\text{to}\; & \sum_{i=1}^{n}x_{ij}=1\;\left(\forall j\in \left\{1,2,\ldots ,n\right\}\right),\\ & \sum_{j=1}^{n}x_{ij}=1\;\left(\forall i\in \left\{1,2,\ldots ,n\right\}\right),\\ & x_{ij}\in \left\{0,1\right\}\;\left(\forall i\in \left\{1,2,\ldots ,n\right\},\forall j\in \left\{1,2,\ldots ,n\right\}\right). \end{array} \end{array}$$ (11)

By transforming the optimization problem (Eq (11)) into the QUBO formulation, we obtain Eq (12).

$$\begin{array}{c} \begin{array}{cc} \text{minimize} & \sum_{i=1}^{n}(1-\sum_{j=1}^{n}x_{ij}{)}^{2}+\sum_{j=1}^{n}(1-\sum_{i=1}^{n}x_{ij}{)}^{2}\\ \;\text{subject}\;\text{to}\; & x_{ij}\in \left\{0,1\right\}\;\left(\forall i\in \left\{1,2,\ldots ,n\right\},\forall j\in \left\{1,2,\ldots ,n\right\}\right). \end{array} \end{array}$$ (12)

In this case, *n*^2^ binary variables are required. The set of solutions explored using quantum annealing has $2^{n^{2}}$ elements. However, the set of feasible solutions has *n*! elements. Therefore, the set of feasible solutions is exponentially smaller than the set of solutions explored using quantum annealing, which makes the search inefficient.

We solved Eq (12) using the quantum annealer D-Wave Advantage and obtained 10000 solutions. In the experiment, we set 10, 30, 50, 70, and 90 μs as the annealing time and used the well-tuned chain strength parameters for each problem size. We describe the tuning of chain strength parameters in Table in S1 Table. The results are summarized in Table 5. In Table 5, **per_f** represents the percentage of feasible solutions. For various annealing time parameters, Table 5 shows that as the problem size *n* increases, the probability of obtaining a feasible solution decreases.

**Table 5:** Probability of obtaining feasible solutions with the tuned chain strength parameters.

n (chain strength)	per_f(10μs)	per_f(30μs)	per_f(50μs)	per_f(70μs)	per_f(90μs)
2 (0.1)	1.0000	0.9998	1.0000	1.0000	0.9999
3 (0.3)	0.9948	0.9981	0.9983	0.9977	0.9986
4 (0.5)	0.8977	0.9245	0.9493	0.9463	0.9584
5 (0.7)	0.4128	0.5176	0.6163	0.6258	0.6309
6 (0.7)	0.2088	0.3021	0.3376	0.3912	0.3907
7 (0.8)	0.0615	0.1212	0.1362	0.1615	0.1659
8 (0.8)	0.0088	0.0182	0.0266	0.0327	0.0318
9 (0.9)	0.0018	0.0021	0.0049	0.0046	0.0069
10 (0.9)	0.0002	0.0001	0.0004	0.0003	0.0005
11 (0.9)	0.0005	0.0007	0.0012	0.0006	0.0011
12 (0.9)	0.0000	0.0000	0.0000	0.0000	0.0000

**per_f** represents the probability of obtaining feasible solutions. The figures in parentheses in **per_f** refer to the annealing time parameters. The figures in parentheses in **n** refer to the chain strength parameters. We used the quantum annealer Advantage_system4.1, taking 10000 samples.

This experiment shows that quantum annealers struggle to obtain feasible solutions even for the example model in Eq (12), whose exact solutions are apparent. Therefore, at least partially, this difficulty will be encountered in solving constrained problems using a quantum annealer. This is also consistent with the result in [33]. Moreover, the penalty terms make the source graph dense and the problem more difficult because the variables in the penalty terms are squared. Because unconstrained optimization problems do not face these difficulties, quantum annealers are more suitable for solving unconstrained optimization problems than constrained optimization problems.

## Numerical experiments and discussion

The break minimization problem is a problem of finding an HA-assignment that minimizes the number of breaks for a given timetable. We conducted two experiments to compare our method based on quantum annealing with two integer programming approaches [16, 18], which exhibited excellent results. In the first experiment, we solved the break minimization problem in an MDRRT using quantum annealing, and two other integer programming approaches presented by Urdaneta *et al*. [18] and Trick [16], respectively. Further, we compared the quality of the solutions and the computational time. In the second experiment, we measured the time it took for the integer programming approach given by Urdaneta *et al*. to reach the objective function value, which the quantum annealer obtained in 0.05 s. To demonstrate the advantage of the sparse source graph, we also conducted the same experiments for the break minimization problem in a DRRT, as well as an MDRRT. Tables 6 and 7 show the results for the first experiment for MDRRTs and DRRTs, respectively. Tables 8 and 9 show the results for the second experiment for MDRRTs and DRRTs, respectively.

**Table 6:** QA vs. IP in MDRRTs.

	QA		IP(Urdaneta)			IP(Trick)		
Teams	Breaks	Time(s)	Breaks	Time(s)	OPTIMAL	Breaks	Time(s)	OPTIMAL
4	6.0	0.05	6.0	0.033974	1.0	6.0	0.039900	1.0
8	19.6	0.05	19.6	0.063883	1.0	19.6	0.288907	1.0
12	38.8	0.05	38.8	0.157146	1.0	38.8	1.951506	1.0
16	66.0	0.05	66.0	0.681240	1.0	66.0	40.549676	1.0
20	106.8	0.05	106.8	3.449914	1.0	106.8	300.039447	0.0
24	161.6	0.05	156.4	52.528646	1.0	-	-	-
28	224.8	0.05	214.0	252.368946	0.2	-	-	-
32	280.4	0.05	267.2	288.295851	0.2	-	-	-
36	368.8	0.05	346.0	300.026792	0.0	-	-	-
40	453.6	0.05	422.4	300.032120	0.0	-	-	-
44	553.6	0.05	520.8	300.024345	0.0	-	-	-
48	663.6	0.05	618.8	300.024338	0.0	-	-	-

**Table 7:** QA vs. IP in DRRTs.

	QA		IP(Urdaneta)			IP(Trick)		
Teams	Breaks	Time(s)	Breaks	Time(s)	OPTIMAL	Breaks	Time(s)	OPTIMAL
4	5.6	0.05	5.6	0.035595	1.0	5.6	0.028979	1.0
8	26.8	0.05	26.8	0.089388	1.0	26.8	0.560439	1.0
12	65.6	0.05	65.6	0.645643	1.0	65.6	10.696434	1.0
16	116.4	0.05	113.2	89.337958	0.8	114.4	300.021179	0.0
20	184.4	0.05	173.6	237.070479	0.4	256.0	300.028045	0.0
24	276.0	0.05	247.6	300.023959	0.0	-	-	-
28	409.6	0.05	362.0	300.023397	0.0	-	-	-

**Table 8:** QA vs. IP(Urdaneta) in MDRRTs.

Teams	Breaks(QA)	Time(Urdaneta)
4	6.0	0.039347
8	19.6	0.062397
12	38.8	0.148171
16	66.0	0.351599
20	106.8	2.837216
24	161.6	8.021498
28	224.8	26.003237
32	280.4	49.271526
36	368.8	84.806435
40	453.6	75.338228
44	553.6	57.133598
48	663.6	6.880923

**Table 9:** QA vs. IP(Urdaneta) in DRRTs.

Teams	Breaks(QA)	Time(Urdaneta)
4	5.6	0.026426
8	26.8	0.055700
12	65.6	0.421455
16	116.4	5.954419
20	184.4	11.851826
24	276.0	9.063769
28	409.6	1.988316

The timetables of the MDRRTs used in the computational experiments were created using the following method.

1. Creating an RRT timetable using the Kirkman method [34].
2. Shuffling the order of the slots in the timetable created in step 1.
3. Concatenating the two identical timetables that are shuffled in step 2.

In this manner, we created five timetables for each number of teams and used common timetables in both computational experiments. The experimental results (Tables 6–9) show the average of the results obtained by solving the break minimization problems defined by the five timetables. In the computational experiments, we compared the following three methods:

- QA: we solve Eq (10) with quantum annealing.
- IP(Urdaneta): we solve Eq (10) using the integer programming approach by Urdaneta *et al*. [18], and we use Gurobi [12] as an integer quadratic programming solver.
- IP(Trick): we solve the problem that equals to the break minimization problem with integer programming approach by Trick [16], and we use Gurobi [12] as an integer linear programming solver.

QA, IP(Urdaneta), and IP(Trick) are abbreviations for each method. Although all three methods were compared in the first experiment (Tables 6 and 7), we compared our quantum annealing approach with the integer programming approach presented by Urdaneta (IP(Urdaneta)) in the second experiment (Tables 8 and 9) because the integer programming approach presented by Urdaneta *et al*. is superior to that of Trick in the first experiment.

The computational environment for IP(Urdaneta) and IP(Trick) is as follows: we used the Gurobi Optimizer(version 9.1.2) on an Intel Core i7–7700HQ 2.80 GHz CPU with four cores and eight threads. We terminated the computation by Gurobi after 300 s because we were required to solve a large number of problems. The parameters for QA are as follows. We used the quantum annealer, D-Wave Advantage. The version of D-Wave Advantage is Advantage_system1.1 and has 5760 qubits. The annealing_time is 50 μs and num_reads is 1000. Therefore, the execution time was 0.05 s. We minor-embed the problem using the method in [35] onto the D-Wave Advantage. We automatically set the chain strength parameters using *uniform torque compensation*, which was developed by D-Wave Systems [36].

The abbreviations in Tables 6 and 7 are as follows: **Teams** is the number of teams, **Breaks** is the average number of breaks in the solution obtained using each method, and **Time** is the average computational time. **OPTIMAL** is the percentage of optimal solutions obtained in 300 s using integer programming approaches. For example, **OPTIMAL** = 0.2 means that one optimal solution out of five was obtained. The abbreviations in Tables 8 and 9 are as follows: **Teams** is the number of teams. **Breaks(QA)** are the average number of breaks in the solution obtained from the quantum annealer. **Time(Urdaneta)** is the average computational time required for IP(Urdaneta) to reach the number of breaks in the solution obtained from the quantum annealer in 0.05 s.

We now explain the results of the first experiment in the MDRRT (Table 6). Both IP(Urdaneta) and IP(Trick) took longer to compute as the number of teams increased. Up to 24 teams, IP(Urdaneta) obtained optimal solutions for all problems within 300 s. However, in problems with more than 28 teams, the obtained solution was either not optimal or the optimality could not be confirmed. The results of IP(Trick) were inferior to those of IP(Urdaneta). IP(Trick) obtained the optimal solutions within 300 s for problems with 16 teams or less. For problems with more than 20 teams, we did not obtain any feasible solutions within 300 s. However, QA obtained optimal solutions for problems with 20 teams or less in 0.05 s. For problems with 24 teams, the difference between the optimal solution and the solution given by QA was only 5.2. For problems with more than 28 teams, the gap between QA and IP(Urdaneta) gradually became wider. We also conducted the same experiment for DRRTs, and the results are summarized in Table 7. Table 7 shows that the gap between Breaks of QA and IP(Urdaneta) is larger in DRRTs, than in MDRRTs, as the number of teams increased. This is because the break minimization problems in MDRRTs are sparser than in DRRTs, as explained in Section “Analysis: Benefits of the sparsity of the problem”. To evaluate how the change in annealing time parameters affects the results, we performed the same additional experiments for QA with various annealing time parameters. When the annealing time was increased, the results (Table 10) showed that the quality of the solutions was improved.

**Table 10:** QA in MDRRTs with various annealing time parameters.

Teams	Breaks(10μs)	Breaks(30μs)	Breaks(50μs)	Breaks(70μs)	Breaks(90μs)	Breaks(110μs)
4	6.0	6.0	6.0	6.0	6.0	6.0
8	19.6	19.6	19.6	19.6	19.6	19.6
12	38.8	38.8	38.8	38.8	38.8	38.8
16	66.4	66.0	66.0	66.0	66.0	66.0
20	108.8	107.6	106.8	106.8	106.8	106.8
24	170.0	162.0	162.4	163.2	159.2	162.4
28	232.4	226.8	226.8	223.6	225.2	222.4
32	296.4	287.2	284.8	279.2	280.4	276.8
36	388.0	378.0	370.8	374.8	364.0	368.8
40	468.0	456.4	442.8	443.2	443.6	440.0
44	578.0	550.0	557.6	550.0	548.8	538.4
48	718.4	669.2	672.8	648.4	654.0	652.0

The figures in parentheses refer to the annealing time parameters. We used the quantum annealer Advantage_system4.1.

We explain the results of the second experiment as follows (Table 8). Because the results of IP(Urdaneta) were superior to those of IP(Trick) in the first experiment (Table 6), we did not conduct the second experiment for IP(Trick). In the second experiment, we measured the time required for IP(Urdaneta) to reach the objective function value, which QA obtained in 0.05 s. **Time(Urdaneta)** in Table 8 shows that as the number of teams increased, the time taken for IP(Urdaneta) to reach the QA’s objective function value also increased, and that the longest time of 84.8 s was required for 36 teams. This demonstrates that quantum annealing has better performance than the solvers in that the solvers take longer to achieve the objective function value of the solution obtained by the quantum annealers. When the number of teams was 40 or more, the time consumed by IP(Urdaneta) gradually shortened. We consider that this is because the quality of the QA solutions deteriorates when there are a large number of teams. As can be observed from **Qubits/Nodes** in Table 3, the number of qubits required to represent a variable increases as the number of teams increases. Because the connectivity between qubits is sparse, multiple qubits are required to represent one variable, which may deteriorate the quality of the solutions. By contrast, the experimental results in a DRRT (Table 9) show that IP(Urdaneta) takes up to 11.8 s to reach the objective function value, which QA obtained in 0.05 s. This result is inferior to that of MDRRTs; therefore, we suggest that the break minimization problem in an MDRRT is more suitable for solving using a quantum annealer, than in a DRRT.

As can be observed from the two experiments, while solving the break minimization problem in an MDRRT, our method that employs the quantum annealer is much faster than integer programming approaches using Gurobi. There are two main reasons for this result. First, as explained in Section “Analysis: Benefits of the sparsity of the problem” and “Analysis: Benefits of no constraints”, the break minimization problem in an MDRRT is sparse and has no constraints; thus, it is suitable to solve using a quantum annealer. Second, as Trick [16] highlighted, the break minimization problem is highly symmetric, and it is difficult to solve using an integer programming approach. In addition, our results differ from those of [9] in that the quantum annealer is faster as the size of the problems increases. In [9], the quantum annealer was faster than Gurobi, but only for small-scale problems.

## Conclusion

In recent years, with the technical development of quantum annealers, extensive research on solving practical combinatorial optimization problems using quantum annealers has been conducted [5–10]. However, researchers struggle to find practical combinatorial optimization problems, for which quantum annealers outperform other mathematical optimization solvers [9, 11]. We determined that the break minimization problem in an MDRRT is a problem for which a state-of-the-art solver such as Gurobi [12] takes longer to achieve the objective function value of the solution obtained by the quantum annealers. We formulated the QUBO of the break minimization problem in an MDRRT by referring to existing studies [18]. Further, we used the two effective existing methods based on integer programming, and our method based on quantum annealing to solve the break minimization problem in an MDRRT, and compared the quality of the solution and computational time. We used Gurobi as an integer programming approach in our experiments. Quantum annealing was able to obtain the exact solution in 0.05 s for the problems with 20 teams, which is a practical size. In the case of 36 teams, it took 84.8 s for the integer programming method to reach the objective function value, which was obtained by a quantum annealer in 0.05 s. The advantage of the method based on quantum annealing is that it is not limited to small-scale problems, which is different from [9]. Our study is also one of the few to compare quantum annealers with commercial solvers, such as Gurobi.

We provided two primary reasons as to why quantum annealers can successfully solve the break minimization problem in an MDRRT. First being, that the break minimization problem in an MDRRT has a sparse structure. We demonstrated that the break minimization problem in an MDRRT can be represented as a 4-regular graph. Such a sparse problem is suitable to solve using quantum annealers. Second being, that the break minimization problem in an MDRRT is unconstrained. We highlighted that the unconstrained optimization problem is suitable to solve because the number of solutions explored using quantum annealing is equal to the number of feasible solutions. These provide an idea about the types of problems that should be solved using a quantum annealer.

## Supporting information

S1 Table Tuning chain strength parameters. We solved Eq (12) using an annealing time of 50μs and considering 10000 samples. This table shows the percentage of feasible solutions for each chain strength parameter. “NaN” indicates that we did not conduct an experiment for this chain strength parameter. Bold letters indicate the highest percentage.
(PDF)

## References

[1] Kadowaki T, Nishimori H. Quantum annealing in the transverse Ising model. Physical Review E. 1998;58(5):5355. doi: 10.1103/PhysRevE.58.5355

[2] Boothby K, Bunyk P, Raymond J, Roy A. Next-generation topology of d-wave quantum processors. arXiv preprint arXiv:200300133. 2020.

[3] D-Wave Systems Inc. QPU Solver Datasheet; 2021. Available from: https://docs.dwavesys.com/docs/latest/doc_qpu.html.

[4] D-Wave Systems Inc. Improved coherence leads to gains in quantum annealing performance; 2019. Available from: https://www.dwavesys.com/media/fbpj1x2v/14-1037a-a_improved_coherence_leads_to_gains_qa_performance.pdf.

[5] Neukart F, Compostella G, Seidel C, Von Dollen D, Yarkoni S, Parney B. Traffic flow optimization using a quantum annealer. Frontiers in ICT. 2017;4:29. doi: 10.3389/fict.2017.00029

[6] Nishimura N, Tanahashi K, Suganuma K, Miyama MJ, Ohzeki M. Item listing optimization for e-commerce websites based on diversity. Frontiers in Computer Science. 2019;1:2. doi: 10.3389/fcomp.2019.00002

[7] Inoue D, Okada A, Matsumori T, Aihara K, Yoshida H. Traffic signal optimization on a square lattice with quantum annealing. Scientific reports. 2021;11(1):1–12. doi: 10.1038/s41598-021-82740-0 33568714 PMC7875976

[8] Negre CF, Ushijima-Mwesigwa H, Mniszewski SM. Detecting multiple communities using quantum annealing on the D-Wave system. Plos one. 2020;15(2):e0227538. doi: 10.1371/journal.pone.0227538 32053622 PMC7018001

[9] Ohzeki M, Miki A, Miyama MJ, Terabe M. Control of automated guided vehicles without collision by quantum annealer and digital devices. Frontiers in Computer Science. 2019;1:9. doi: 10.3389/fcomp.2019.00009

[10] Stollenwerk T, O’Gorman B, Venturelli D, Mandra S, Rodionova O, Ng H, et al. Quantum annealing applied to de-conflicting optimal trajectories for air traffic management. IEEE transactions on intelligent transportation systems. 2019;21(1):285–297. doi: 10.1109/TITS.2019.2891235

[11] O’Malley D, Vesselinov VV, Alexandrov BS, Alexandrov LB. Nonnegative/binary matrix factorization with a d-wave quantum annealer. PloS one. 2018;13(12):e0206653. doi: 10.1371/journal.pone.0206653 30532243 PMC6287781

[12] Gurobi Optimization, LLC. Gurobi Optimizer Reference Manual; 2021. Available from: https://www.gurobi.com.

[13] Cplex, IBM ILOG. V12. 1: User’s Manual for CPLEX. International Business Machines Corporation. 2009;46(53):157.

[14] Ribeiro CC. Sports scheduling: Problems and applications. International Transactions in Operational Research. 2012;19(1-2):201–226. doi: 10.1111/j.1475-3995.2011.00819.x

[15] Rasmussen RV, Trick MA. Round robin scheduling–a survey. European Journal of Operational Research. 2008;188(3):617–636. doi: 10.1016/j.ejor.2007.05.046

[16] Trick MA. A schedule-then-break approach to sports timetabling. In: International Conference on the Practice and Theory of Automated Timetabling. Springer; 2000. p. 242–253.

[17] Régin JC. Minimization of the number of breaks in sports scheduling problems using constraint programming. DIMACS series in discrete mathematics and theoretical computer science. 2001;57:115–130.

[18] Urdaneta HL, Yuan J, Siqueira AS. Alternative Integer linear and Quadratic Programming Formulations for HA-Assignment Problems. Proceeding Series of the Brazilian Society of Computational and Applied Mathematics. 2018;6(1). doi: 10.5540/03.2018.006.01.0311

[19] Elf M, Jünger M, Rinaldi G. Minimizing breaks by maximizing cuts. Operations Research Letters. 2003;31(5):343–349. doi: 10.1016/S0167-6377(03)00025-7

[20] Miyashiro R, Matsui T. Semidefinite programming based approaches to the break minimization problem. Computers & Operations Research. 2006;33(7):1975–1982. doi: 10.1016/j.cor.2004.09.030

[21] Suzuka A, Miyashiro R, Yoshise A, Matsui T. The home–away assignment problems and break minimization/maximization problems in sports scheduling. Pacific Journal of Optimization. 2007;3:113–33.

[22] Nemhauser GL, Trick MA. Scheduling a major college basketball conference. Operations research. 1998;46(1):1–8. doi: 10.1287/opre.46.1.1

[23] Schreuder JA. Combinatorial aspects of construction of competition Dutch professional football leagues. Discrete Applied Mathematics. 1992;35(3):301–312. doi: 10.1016/0166-218X(92)90252-6

[24] Rasmussen RV. Scheduling a triple round robin tournament for the best Danish soccer league. European Journal of Operational Research. 2008;185(2):795–810. doi: 10.1016/j.ejor.2006.12.050

[25] Ribeiro CC, Urrutia S. Scheduling the Brazilian soccer tournament with fairness and broadcast objectives. In: International Conference on the Practice and Theory of Automated Timetabling. Springer; 2006. p. 147–157.

[26] Kirkpatrick S, Gelatt CD, Vecchi MP. Optimization by simulated annealing. science. 1983;220(4598):671–680. doi: 10.1126/science.220.4598.671 17813860

[27] Miyashiro R, Matsui T. Round-robin tournaments with a small number of breaks. Department of Mathematical Informatics, The University of Tokyo, Mathematical Engineering Technical Reports METR. 2003;29:2003.

[28] De Werra D. Scheduling in sports. Studies on graphs and discrete programming. 1981;11:381–395. doi: 10.1016/S0304-0208(08)73478-9

[29] D-Wave Systems Inc. dwave-system Documentation Release 1.6.0; 2021. Available from: https://docs.ocean.dwavesys.com/_/downloads/system/en/stable/pdf/.

[30] Hamerly R, Inagaki T, McMahon PL, Venturelli D, Marandi A, Onodera T, et al. Experimental investigation of performance differences between coherent Ising machines and a quantum annealer. Science advances. 2019;5(5):eaau0823. doi: 10.1126/sciadv.aau0823 31139743 PMC6534389

[31] Koopmans TC, Beckmann M. Assignment problems and the location of economic activities. Econometrica: journal of the Econometric Society. 1957; p. 53–76. doi: 10.2307/1907742

[32] Dantzig G, Fulkerson R, Johnson S. Solution of a large-scale traveling-salesman problem. Journal of the operations research society of America. 1954;2(4):393–410. doi: 10.1287/opre.2.4.393

[33] Kuramata M, Katsuki R, Nakata K. Larger Sparse Quadratic Assignment Problem Optimization Using Quantum Annealing and a Bit-Flip Heuristic Algorithm. In: 2021 IEEE 8th International Conference on Industrial Engineering and Applications (ICIEA). IEEE; 2021. p. 556–565.

[34] Kirkman TP. On a problem in combinations. Cambridge and Dublin Mathematical Journal. 1847;2:191–204.

[35] Cai J, Macready WG, Roy A. A practical heuristic for finding graph minors. arXiv preprint arXiv:14062741. 2014.

[36] D-Wave Systems Inc. D-Wave System Documentation: Uniform Torque Compensation; 2018. Available from: https://docs.ocean.dwavesys.com/projects/system/en/latest/reference/generated/dwave.embedding.chain_strength.uniform_torque_compensation.html#dwave.embedding.chain_strength.uniform_torque_compensation.

